# The healthcare system in Sweden

**DOI:** 10.1007/s10654-025-01226-9

**Published:** 2025-05-19

**Authors:** Jonas F. Ludvigsson, David Bergman, Catharina Ihre Lundgren, Kristina Sundquist, Jean-Luc af Geijerstam, Anna H. Glenngård, Marie Lindh, Johan Sundström, Johan Kaarme, Jialu Yao

**Affiliations:** 1 Department of Medical Epidemiology and Biostatistics, Karolinska Institutet, Stockholm, SE-171 76 Sweden; 2 Department of Paediatrics, Örebro University Hospital, Örebro, Sweden; 3 Department of Medicine, Columbia University College of Physicians and Surgeons, New York, NY USA; 4 Department of Molecular Medicine and Surgery, Karolinska Institutet, Stockholm, Sweden; 5 Department of Breast, Endocrine Tumors, and Sarcoma, Karolinska University Hospital, Stockholm, Sweden; 6 Center for Primary Healthcare Research, Department of Clinical Sciences Malmö, Lund University, Lund, Sweden; 7 The Swedish Agency for Health and Care Services Analysis, Stockholm, Sweden; 8 Department of Business Administration, Lund University School of Economics and Management, Lund, Sweden; 9 The Swedish Stomach and Bowel Association, Stockholm, Sweden; 10 Clinical Epidemiology Unit, Department of Medical Sciences, Uppsala University, Uppsala, Sweden; 11 The George Institute for Global Health, University of New South Wales, Sydney, Australia; 12 Swedish Association of Local Authorities and Regions, Stockholm, Sweden; 13 Department of Paediatrics, Uppsala University Hospital, Uppsala, Sweden

**Keywords:** Health care, Sweden, Swedish, Epidemiology, Public Health

## Abstract

The Swedish population is characterized by high life expectancy and low avoidable mortality rates. This review outlines the Swedish healthcare system, which offers universal access to all residents and has a long tradition of reforms for social equity. Responsibility for healthcare is shared between the state, the regions, and the municipalities. The Ministry of Health and Social Affairs provides the overall healthcare framework; additionally, several governmental agencies are directly involved in healthcare and public health initiatives. The 21 regions organize, finance, and provide most primary, secondary, and tertiary care, as well as health information channels. Resources for primary care are less plentiful than in many other countries. The 290 municipalities deliver care to elderly people and those with functional impairment. The Swedish healthcare system is primarily tax-funded, with 86% of total healthcare expenditures from public expenses and < 1% from voluntary health insurance. The gross domestic product (GDP) share of healthcare expenditures, 10.5% in 2022, is above the EU average. The level of unmet needs in the population is low, due to universal coverage and caps on user charges except for dental care. Sweden’s healthcare system performs well on care quality and patient satisfaction, but suffers from workforce shortage and care fragmentation. Limitations in care coordination can be attributed to a siloed digital infrastructure and care governance, a low number of hospital beds per capita, and a compensation system that often does not incentivize coordination. Despite these challenges, life expectancy is high and avoidable mortality rates are low in Sweden.

## Introduction

Sweden is a country in the north of Europe, bordering Norway, Finland, and Denmark. As of October 2024, about 10.6 million people are living in Sweden. Of the total population, 49.7% are female, the median age is 41.0 years, and individuals under 18 years and those over 65 years each comprise about 20% [1]. Swedish citizens comprise 92% of the total population, and 1 in 5 residents were born abroad. The largest foreign-born groups include individuals of Syrian, Iraqi, Finnish, Polish, and Iranian origins (each < 0.5%) [1]. The official main language is Swedish, but English literacy is high across the population [2].

**Fig. 1:**
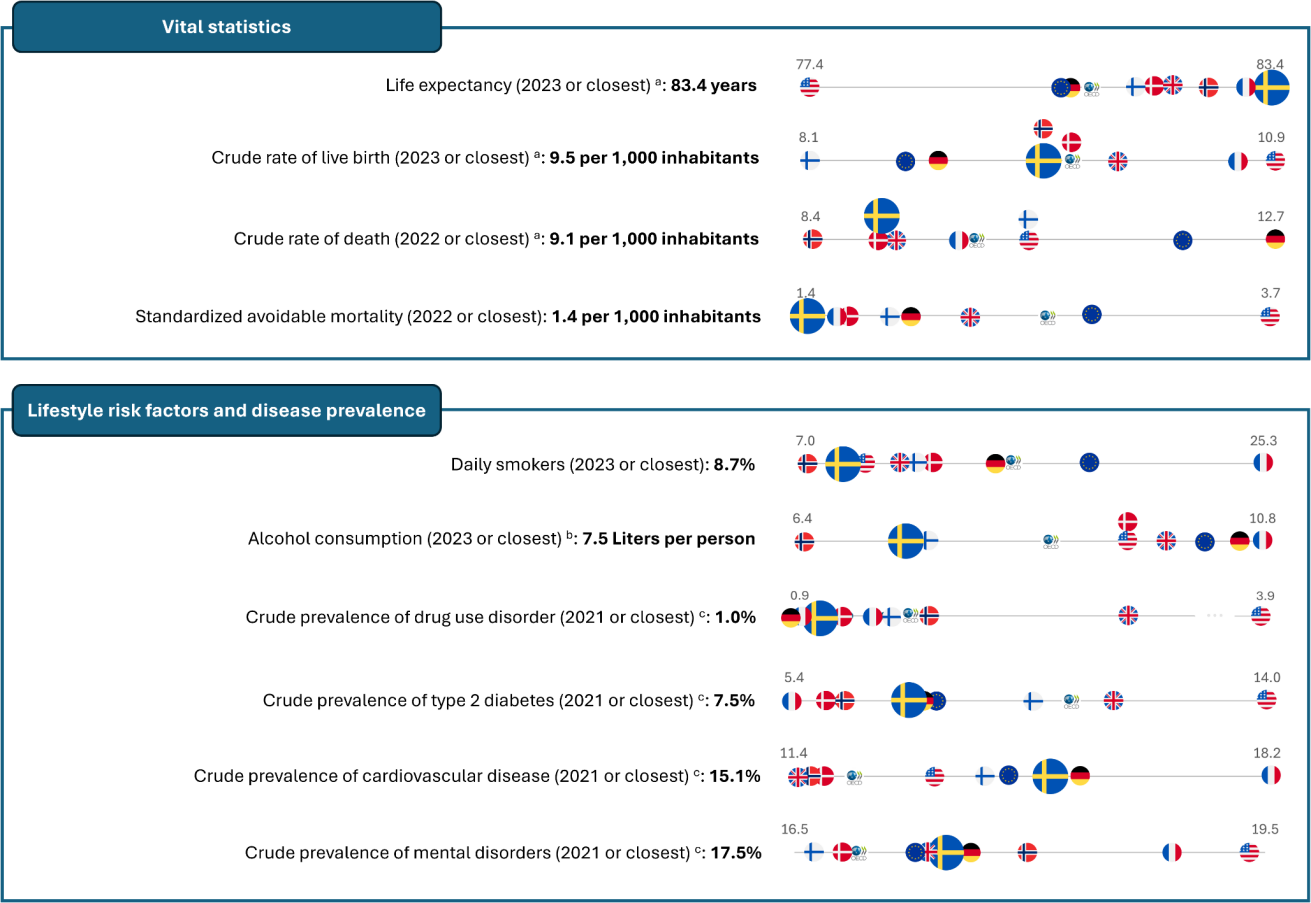
Vital statistics and the prevalence of selected lifestyle risk factors and diseases. Values for Sweden are marked in bold. Values for Sweden are compared with seven countries including Denmark, Finland, France, Germany, Norway, the United Kingdom, and the United States, as well as the unweighted average across the Organization for Economic Co-operation and Development (OECD) and the European Union (EU), represented by the flag of each economic entity. Data source, if not stated otherwise: *OECD Data Explorer (*https://www.oecd.org/en/data/datasets/). ^a^ Missing values were filled with the data from the World Bank Open Data (https://data.worldbank.org/). ^b^ Missing values were filled with the data from the Eurostat (https://ec.europa.eu/eurostat). ^c^ Data from the Global Burden of Disease Study (https://ghdx.healthdata.org/)

The Swedish population enjoys a high material standard (with a material deprivation rate lower than its Nordic counterparts), a high life expectancy, and a low rate of avoidable mortality (see Fig. 1 for a cross-country comparison) [3, 4]. Measured by health-adjusted life expectancy at birth and healthy life years, Sweden is ranked fourth and eighth among countries of the European Union (EU) [5], respectively. Infant mortality is low (2.5 per 1000) and Sweden is among the top three countries in the world for several other measures, such as children-under-five mortality [6].

Sweden established universal healthcare in the latter half of the 20th century and has since continuously adapted to evolving healthcare needs of its population [7]. We aim to review and describe the Swedish healthcare system and outline some recent reforms. This work seeks to provide context for clinicians and researchers, both domestically and internationally, and to interpret results derived from Swedish healthcare data.

## Leadership and governance

Sweden has a decentralized healthcare system. Priorities in health and medical care are guided by three basic ethical principles, as stated in the Government Bill 1996/97:60 [8]: (i) the human value principle: all human beings have an equal entitlement to dignity and should have the same rights, regardless of their socioeconomic status in the community; (ii) the need and solidarity principle: individuals are prioritized by their needs for health and medical care; and (iii) the cost-effectiveness principle, which is subordinate to the other two principles: when a choice for medical care has to be made, one should strive for a reasonable balance between costs and effects (in terms of improved health and quality of life).

### The legislative framework for Swedish healthcare

The Health and Medical Services Act of 2017 (SFS 2017:30) constitutes the central legal framework governing healthcare [9]. This act is a revision of the 1982 Health and Medical Service Act (SFS 1982:763) [9], and sets the legal basis of the contemporary Swedish healthcare system [10]. The Health and Medical Service Act lays out overarching healthcare objectives and delineates the responsibilities of different healthcare stakeholders, particularly during the restructuring of Swedish healthcare system. This national framework law underlines that healthcare is fundamental to an individual’s life and well-being. It mandates the regions and municipalities’ provision of care to the entire population while upholding high standards, hygienic practices, and equitable access to quality care. Human dignity and equity aspects are emphasized: care must not be given against the will of the patient, and healthcare should be planned and performed after consulting with the patient; priority should be given to patients with the greatest needs (i.e., vertical equity) while ensuring that access to care is not influenced by the individual’s socioeconomic factors such as income, sex, age, nationality, and culture (i.e., horizontal equity).

The provision of healthcare is generally regulated under the Patient Act (SFS 2014:821) [11]. This Act strengthens and clarifies that patient needs are the central driving force behind healthcare decisions. The Patient Act (SFS 2014:821) defines patients’ right to be informed about their conditions, treatment options, and to participate in treatment decisions [11]. Sometimes, this requires written information or an interpreter to ensure patient comprehension. Patients also have the right to obtain a second opinion when facing serious or life-threatening conditions. Regional and municipal entities are obligated to provide means for ensuring patient integrity, self-determination, and engagement [10].

As a means of communication, Sweden offers web- and phone-based, around-the-clock support through service 1177 (named after its dialing numbers), which is provided in many different languages. Web-based 1177 services grew rapidly during the COVID-19 pandemic when the public sought online venues for healthcare advice.

There are also laws targeting more specific areas of care provision, examples of which are listed in the following paragraphs. The Patient Safety Act (SFS 2010:659) stipulates healthcare providers’ responsibility to protect patients from healthcare-related adverse events [12]. This Act regulates licensed professions in healthcare and procedures for dealing with patient complaints. The Health and Social Care Inspectorate (*Inspektionen för vård och omsorg*, *IVO*) is the supervisory authority for healthcare and healthcare practitioners.

The Act on Coordinated Discharge (SFS 2017:612) coordinates healthcare and social services between regional and municipal providers (see section “**The regions and the municipalities**” for details) [13]. It aims to reduce discharge delays from inpatient care and mandates individual care planning before formal discharge. The Act stipulates that municipalities are obliged to pay a fee to its region starting three days after a hospitalized patient has been deemed to be medically ready for discharge [13].

The Swedish parliament (*Riksdag*) promulgated the Freedom of Choice Act (SFS 2008:962) to enhance person-centeredness by empowering patients to choose their healthcare provider and to stimulate care quality improvement through competition [14]. The Act stipulates that individuals can choose their care providers among those approved by the local government. The Act took effect for primary care in 2010 (choice of primary care center) and specialized outpatient care in 2015 (the latter fueled by the nationwide growth of private digital healthcare in 2016, most often tax-financed through agreements) [10]. Specifically, regions must follow the Freedom of Choice Act for primary and specialist outpatient care (although *direct* access to specialist care varies across regions). In cases when the freedom of choice may be hampered by insufficient access and unequal distribution of health resources, patients could seek care in other regions, and cross-regional compensations are jointly agreed upon [10]. On the other hand, municipalities’ long-term care or social services are not bound by freedom of choice. In 2023, 159 of 290 municipalities had adopted a freedom-of-choice system in at least one social service area [15]. As an extension, patients could also be reimbursed for planned medical treatment in another EU/EEA country or Switzerland after prior authorization (*förhandstillstånd*) or prior decision (*förhandsbesked*) from the Social Insurance Agency (*Försäkringskassan*) [16] if certain conditions are fulfilled, (for cases seeking prior authorization) e.g., the specific care is included in the Swedish universal healthcare system, the provider in the country of care is affiliated with the public healthcare system, and the current waiting time in Sweden is not medically justifiable.

### Organizations

In Sweden, healthcare responsibilities are tripartite, shared among the national government, the 21 regional authorities, and the 290 municipalities.

### The state

The Swedish parliament (*Riksdag*), as the representative of the people of Sweden, holds the legislative power [10]. The Ministry of Health and Social Affairs (*Socialdepartementet*) is responsible for all issues concerning social welfare. The parliament and the Ministry of Health and Social Affairs share responsibility for comprehensive healthcare governance, encompassing legislative frameworks and budgetary appropriations. Such appropriations include both general and designated grants, such as those for mental health services and cancer screening programs [10]. The overall budgetary appropriations account for about 16% of the regions’ total revenue and 21% of the municipalities’.

The Ministry of Social Affairs has several national agencies that are directly involved in healthcare and public health (Table 1) [10]. These agencies develop evidence or support research on the health of the general population and the performance of the healthcare system; regulate and supervise the safety and quality of services and medical products; decide on reference prices for medical products; and coordinate health system reform. Moreover, they are tasked with preparing for crises such as the COVID-19 pandemic.

**Table 1:** Authorities under the ministry of social affairs that are directly related to healthcare and public health

Name in Swedish (Commonly used abbreviation, if any)	Name in English (Commonly used abbreviation, if any)	Main responsibility
Socialstyrelsen	The National Board of Health and Welfare (NBHW)	National knowledge authority in health and care • Central advisory and regulatory agency in healthcare and social services. • Issues standards and guidelines. • Supports knowledge dissemination. Maintains health registers and official statistics. • Licenses healthcare staff • Directs advisory and decision-making bodies on issues regarding, e.g., judicature (the Legal Council, *Rättsliga rådet*), ethics (the Ethics Council, *Etiska rådet*), and specialized care (the Board for National Highly Specialised Care, *Nämnden för nationellt högspecialiserad vård*) • Strengthens society and healthcare system preparedness for emergencies. • Maintains health registers (49–54) • Carries out comparisons between regions and hospitals on healthcare quality, so-called “open comparisons” (*Öppna jämförelser*)
Inspektionen för vård och omsorg (IVO)	The Health and Social Care Inspectorate	• Oversees activities and personnel involved in healthcare and social services • Determines permission for private providers of social services and care services such as blood services, tissue banks, circumcision, and syringe exchange services.
Hälso- och sjukvårdens ansvarsnämnd (HSAN)	The Medical Responsibility Board	• Examines authorization issues of licensed health and healthcare practitioners. • Decides on disciplinary measures for complaints and possible malpractice or questions notified by IVO or practitioners.
Tandvårds- och Läkemedelsförmånsverket (TLV)	The Dental and Pharmaceutical Benefits Agency	• Evaluates medical products (e.g., medications, medical devices, medical device consumables) for pricing and subsidy by deciding on national drug benefit schemes, pharmaceutical market regulations, the trading margins of pharmacies, and the high-cost protection (*högkostnadsskyddet*).
Statens Beredning för Medicinsk och Social Utvärdering (SBU)	The Agency for Health Technology Assessment and Assessment of Social Services	• Independently assesses medical products and medical interventions by summarizing and communicating related evidence. • Identifies knowledge gaps in healthcare.
Läkemedelsverket (LV)	The Medical Products Agency (MPA)	• Regulates and supervises the development, manufacturing, and sale of pharmaceutical products and medical devices.
Folkhälsomyndigheten (FOHM)	The Public Health Agency (PHA)	• Promotes good and equal health, prevents diseases and injuries, works for effective infection control, and protects the population from various health threats. Particular attention will be paid to the groups most at risk of suffering from ill health. • Provides knowledge-based regulations, recommendations, and guidance for healthcare practitioners about public health. • Organizes public health programs such as vaccination and antibiotic use stewardship.
Myndigheten för vård- och omsorgsanalys (short name: Vård- och omsorgsanalys)	The Agency for Health and Care Services Analysis	• Evaluates and reviews the effectiveness of healthcare and social services from a person-centered perspective
Forskningsrådet för hälsa, arbetsliv och välfärd (FORTE)	The Research Council for Health, Working Life and Welfare	• Initiates and financially supports basic or need-based research related to health, working life, and welfare.
E-hälsomyndigheten	The e-Health Agency	• Coordinates the government’s e-health initiatives and monitors developments in the e-health field. • Facilitates information exchange within health and care, such as the digitalization of prescriptions.
Försäkringskassan	The Social Insurance Agency (SIA)	• Provides financial security for illness, disability, travel, and parental care.
Myndigheten för Delaktighet (MFD)	The Swedish Agency for Participation	• Facilitates the implementation of the disability policy.

The government can sign agreements with the regions via the Swedish Association of Local Authorities and Regions (SALAR; Swedish: *Sveriges Kommuner och Regioner*) and other organizations to govern healthcare. Such agreements include, e.g., improved delivery care and healthcare for women, person-centered care (including targeted investments in ambulance care, improved management of antibiotics, and support of the Swedish National Quality Registers [17], pharmaceutical supplies in times of pandemics, financial support to cover costs for prescriptions, equal cancer care, shorter waiting time to healthcare (e.g., the “healthcare guarantee” or “waiting time guarantee” *(“vårdgaranti”*)), suicide prevention and work to improve psychiatric health, and (previous) reimbursement for COVID-19 vaccinations. These agreements have served as a governmental mechanism to influence healthcare, thus supplementing existing legislation.

### The regions and the municipalities

Although subject to statewide regulatory frameworks, the 21 regions and 290 municipalities exercise considerable autonomy in healthcare administration. Regional and municipal governments set priorities based on population needs, decide on the types of care services they provide and the reimbursement mechanisms, and are responsible for the quality of care they provide. Decentralized healthcare is further illustrated through taxing rights. Regional and municipal governments possess autonomous authority to impose income taxes for healthcare funding.

Most primary and specialized care, whether concerning somatic or psychiatric conditions or in the inpatient or outpatient settings, is financed, organized, and provided by the regions. However, the proportion of private primary care providers varies across regions, with Region Stockholm having the highest share (71% in 2023) [18]. Each region has a political board (previously called the county council or “*landsting*” in Swedish). The regional political boards are responsible for specialized in- or outpatient care, primary care (including pediatric and maternity care) provided in the primary care centers, and care by general practitioners in ordinary and special housing. Collectively, the regions have about 1,200 primary care centers, 7 university (regional) hospitals, 20 county hospitals (*Länssjukhus*), and 40 smaller county hospitals (*Länsdelssjukhus*). Of all these hospitals, approximately 60 offer emergency care [10].

Following a health needs assessment, municipalities provide healthcare and social services to the elderly people and those with functional impairment. The municipalities cover home-based care (e.g., “*dagverksamhet*,” with exception for municipalities in Region Stockholm) or residential care for the elderly (i.e., nursing homes that are short-term “*korttidsboende*” or long-term “*särskilt boende/sä-bo*”) and provide social services for children and adults with functional impairments (The Act Concerning Support and Service for People with Certain Functional Impairments, SFS 1993:387) [19]. In addition, municipalities within most regions are responsible for patients’ home-based rehabilitation after discharge, which includes coordinated medical care and daily activity support. Municipal healthcare services were provided to approximately 414,000 individuals in 2023; 83% were aged ≥ 65 years, with roughly half being women in this age group [20].

The region’s hospitals and primary care centers manage conditions that do not require highly specialized care for residents in their catchment area. Municipalities become economically responsible for hospitalized patients ready to be discharged after three days of physician-suggested discharge. This “responsibility relay” aims at shorter inpatient episodes to reduce adverse events, such as hospital-acquired infections. However, recent years have seen an increase in unmet needs for rehabilitation care, particularly in municipalities with limited human and physical resources for long-term care [10].

**Fig. 2:**
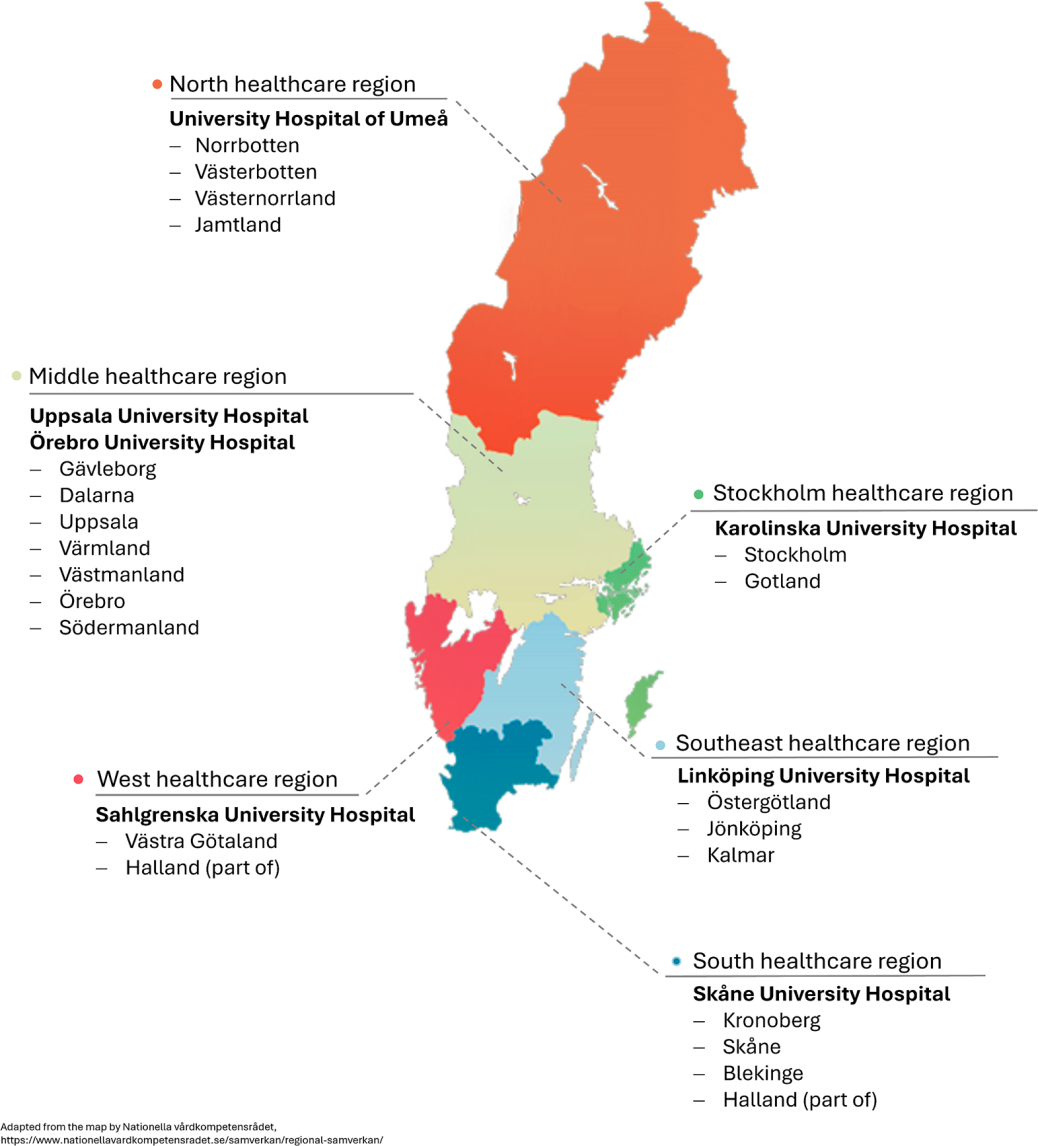
Healthcare regions and distribution of university hospitals

Regions collaborate extensively within six larger healthcare regions (see Fig. 2 for the composing regions of each healthcare region as well as the distribution of university hospitals). Within each larger healthcare region, there is at least one university hospital. These university hospitals are multifaceted, serving as regional hospitals (sometimes having multiple sites), specialist hospitals, teaching hospitals, and research hospitals. Specifically, university hospitals offer care for residents of the larger healthcare region, perform highly advanced diagnostics and cutting-edge care techniques for complicated or rare cases in a larger catchment area, and deliver basic and advanced levels of education to healthcare practitioners (regulated by the Agreement on Medical Education and Research, *Avtal om Läkarutbildning och Forskning*, between the Swedish government and the seven regions where university hospitals are located at). In Sweden, university hospitals are run by the regions and are separate from the universities, which are owned by the state.

Despite the high governing autonomy and taxation independence, all municipalities and regions in Sweden are collectively represented by SALAR. SALAR is a members’ organization that negotiates with the state for the interest of local government and local democracy. It is also the central employers’ association for staff employed by the regions and municipalities (see later section “**Health workforce**” for details) and provides expertise on healthcare issues.

To harmonize healthcare guidelines across the country, the six healthcare regions, along with SALAR, have initiated a collaboration denoted the national system for knowledge-driven management (*kunskapsstyrning hälso- och sjukvård)*.

### Other actors in healthcare

In addition to the state government and the regional and municipal authorities, healthcare in Sweden is influenced by a number of interest organizations. The Swedish Society of Medicine (*Svenska läkaresällskapet*) is an independent and professional organization for physicians, medical students, and allied healthcare professionals. It promotes research, education, and quality assurance in medicine, and serves as a platform for ethical discussions. The society has more than 50 associate societies and some 34,000 members.

The Swedish Medical Association (*Sveriges läkarförbund*) is a trade union with 58,000 physicians and medical students. It focuses on salaries, the working environment, leadership, and entrepreneurship in healthcare. Nurses, midwives, biomedical scientists, and radiographers are represented by the Swedish Association of Health Professionals (*Vårdförbundet*). They organize some 114,000 individuals, constituting 80% of the relevant workforce. Pharmacists are represented by the Swedish Pharmacist Association (*Sveriges Farmaceuter*). There are trade associations that include the Association for Private Care Providers, Almega (*Vårdföretagarna*), which represents private employers; the Swedish Pharmacy Association (Sveriges Apoteksförening), which represents Swedish pharmacies; and the Research-Based Pharmaceutical Industry in Sweden (*LIF*,* De forskande läkemedelsföretagen*) for research-based pharmaceutical industry in Sweden. LIF produces “FASS” (*Farmaceutiska Specialiteteri Sverige*), an information service that contains updated and high-quality drug information for healthcare professionals and the public. FASS has been developed from a printed book to a website (https://www.fass.se/LIF/startpage) and is currently the second most used website in healthcare after the information website 1177.se. FASS covers drug indications, side effects, and additional product information including the dispensation routes, product sizes, and (sometimes) approximate prices.

Diseases with large patient groups usually have their own patient organizations. Among such organizations are the Swedish Rheumatism Association, the Swedish Diabetes Association, and the Asthma and Allergy Association. These organizations have dual roles for the patients they represent and the wider community. They help members manage everyday life and disease consequences, influence policies and public healthcare, and promote targeted research in their field.

Pediatricians primarily serve young children and those with acute or chronic health issues, whereas other children typically begin care with a general practitioner [20] (Table 2). A description of the Swedish pediatric healthcare system has been provided by Wettergren et al. in 2016 [20].

**Table 2:** Healthcare trajectories of four case examples

Girl, 6 years old with suspected asthma	A female, 37 years old, felt a lump in her left breast	Male, 40 years old with suspected IBD	Female, 82 years old, difficulties breathing
If not urgent, the family will first contact the local primary healthcare center, where a general practitioner will examine the patient. If the girl is deemed to need asthma medication, she will be referred to the pediatric outpatient clinic for further investigation and lung function tests. There, she will meet a specialized pediatric asthma nurse who will carry out spirometry, while a pediatrician or a pediatric allergist will take a detailed medical history and conduct a physical examination. Once the asthma diagnosis is confirmed, the girl will be put on asthma long-term control and reliever medication. If she has moderate to severe symptoms, she will be followed up once or twice a year in the pediatric outpatient clinic. If the girl is deemed to have mild asthma, she will be referred to primary care for follow-up.	The patient has no family history of previous lumps or breast cancer and calls her general practitioner. A physical examination shows a suspicious lump, and she starts a fast track for suspected cancer care (“SVFF”). She receives an appointment for mammography within a week. This shows a 2 cm lump, and she is called back to do a core biopsy and ultrasound. Five days later, she will see a breast surgery specialist, who informs her of her breast cancer diagnosis. A multidisciplinary conference will discuss her case within 4 days, and within 3 weeks, she will go through a breast-conserving operation and a sentinel node biopsy in the axilla. Depending on tumor characteristics and treatment advice, follow-up will occur in specialist care for at least 5 years.	The patient will first contact his local primary healthcare center if it is not extremely urgent. In case symptoms and initial examinations suggest that he may suffer from cancer, the patient will follow a fast-track of standardized cancer care (”SVFF”). The general practitioner will investigate the patient and obtain additional laboratory tests if non-cancer disease is suspected. If IBD is suspected, the patient will be referred to an internal medicine or gastroenterology department. On average, within a few months, the patient will see a gastroenterologist/internist and undergo an endoscopy. Medication will be started, and the patient will be followed up at a specialist clinic after 1-2 months and after another 6–12 months. Routine visits may then be scheduled every 1–2 years, and patients doing well may be remotely monitored and only seen on-demand (i.e., when they progress or have questions).	The patient’s husband calls 112, and an ambulance arrives within 10 min. The patient has had an annoying cough for a few days. Her oxygen saturation is 92% and she receives extra oxygen. She has a previous history of asthma and had a myocardial infarction 10 years ago. She is otherwise healthy for her age. When arriving at the emergency department, blood tests and an ECG are performed, and a CT scan of her chest to rule out thromboembolism or ischemic heart disease. However, an intrathoracic goiter on the right side of the neck is visualized, and while her trachea has a normal range, it deviates to the left. During her stay at the emergency department, she recovers well, and her breathing normalizes. Follow-up can be done in primary care. A referral will then be sent to a specialist to discuss surgery for her goiter.

IBD, inflammatory bowel disease; ECG, electrocardiogram; CT, computed tomography

## Financing

**Fig. 3:**
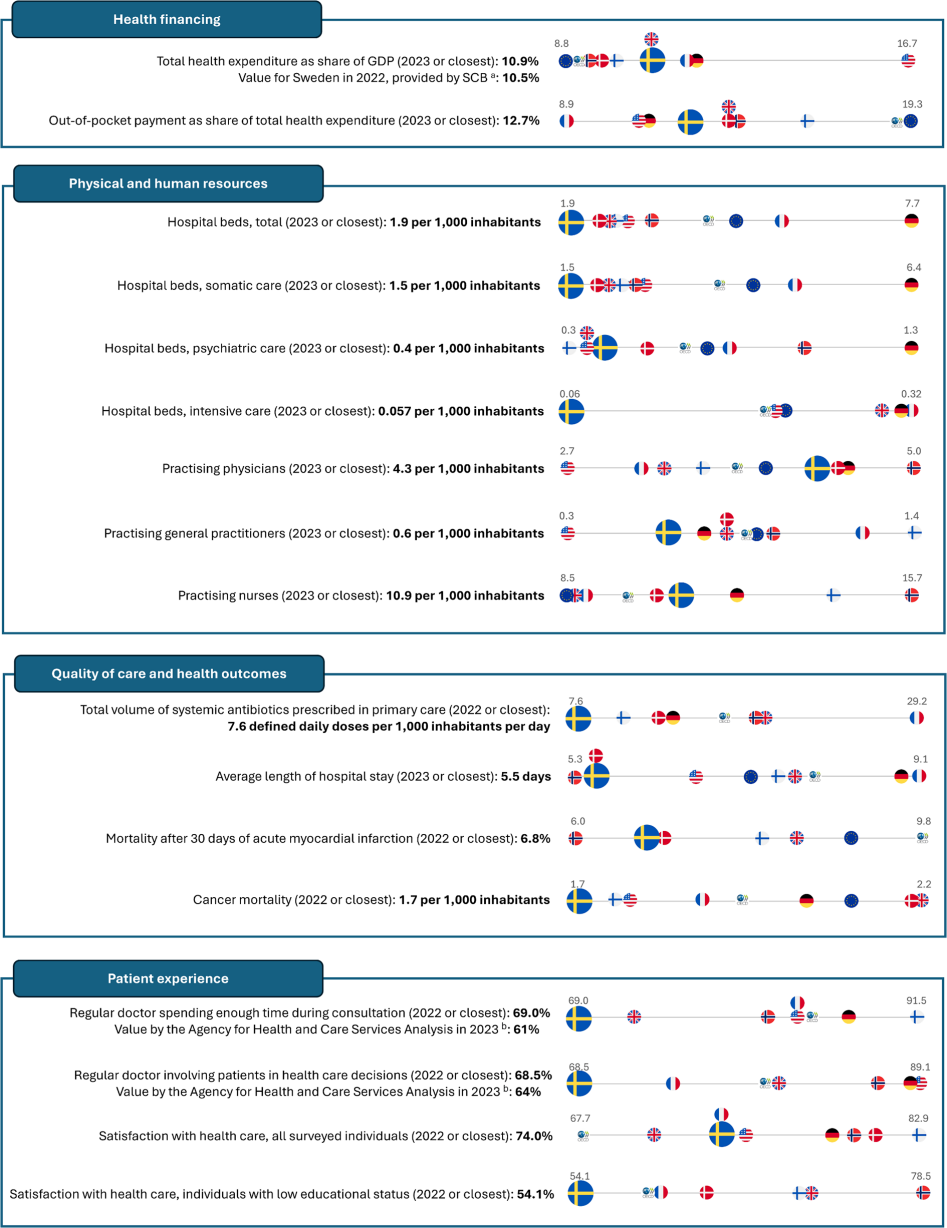
Indicators related to health financing, physical and human resources, quality of care and health outcomes, and patient experience. Values for Sweden are marked in bold. Values for Sweden are compared with seven countries including Denmark, Finland, France, Germany, Norway, the United Kingdom, and the United States, as well as the unweighted average across the Organization for Economic Co-operation and Development (OECD) and the European Union (EU), represented by the flag of each economic entity. Data source, if not stated otherwise: OECD Data Explorer (https://www.oecd.org/en/data/datasets/oecd-DE.html). ^a^ Values published by SCB (Statistikmyndigheten SCB [Statistics Sweden]) could be found at https://www.scb.se/. ^b^ Values from Vården ur befolkningens perspektiv (PM 2024:3) [Care from the population’s perspective (PM 2024:3)] (https://www.vardanalys.se/digital-publikation/varden-ur-befolkningens-perspektiv/)

Healthcare in Sweden is primarily tax-funded. Sweden spends about 10.5% of its GDP on healthcare (most recent data from 2022) [21]. This level of share positions Sweden among the leading EU countries, with Germany, France, and Denmark as its main competitors [22] (see Fig. 3 for a cross-country comparison of health financing). Since 2011, Sweden has included elderly care and care for people with functional impairment when calculating health expenditure, and its share in GDP has not changed much since. Among countries outside the EU, for instance Norway and Switzerland spend more on healthcare than Sweden.

Public expenditures constitute 86% of aggregate Swedish healthcare spending; this includes 2% from the state, 59% from regional or region-affiliated entities, and 25% from municipalities [23]. Although local tax revenues are still the main source of public funding [10], the past decade has seen general increases in the share of healthcare funded by national government grants on both regional and municipal levels [10].

In 2023, healthcare consumed roughly 88% of the total expenditure the regions bear [24]. Among the region’s total expenditure, some 47% is consumed by specialized (hospital-based) somatic care and about 16% by primary care. Specialized psychiatric care and dental care represent 7% and 3%, respectively, with other healthcare making up a few percent of the costs [24]. It is more difficult to estimate the cost of municipal healthcare, because costs are often shared between actual healthcare and general social care of the elderly (especially when mixed with support for daily activities such as bathing, eating, dressing, and grocery shopping). However, by one estimate, municipalities spend approximately 30% of their total expenditure on caring for the elderly and individuals with functional impairments [24].

There are various payment models for different services, and they are set at the local government’s discretion. Primary care centers receive reimbursement from regions based primarily on per-capita rates adjusted to anticipated healthcare and sociodemographic needs (see the next paragraph for details). Payment mechanisms are more heterogeneous for specialized somatic and psychiatric care. Over the past decade, the fixed global budget has regained its dominance, while case-based compensations (i.e., those based on diagnosis-related groups or care episodes) are used selectively for public-funded care by private providers and several outpatient services. Activity-based compensations (e.g., fee-for-service) are applied to dental care and digital consultations; however, their application in primary care remains limited [10].

A typical capitation starts with an adult (18–64 years) as the reference, and funding is added for children and older adults or if the individual is unemployed, born outside Europe, lives alone with children aged < 18 years, or has a lower education. Additional funding can also be granted when a primary healthcare center has a high number of asylum seekers or is located remotely from the nearest hospital (since, in such areas, more inhabitants will frequent primary care rather than the hospital). For instance, in Region Örebro, capitation costs vary by 40% between primary healthcare centers (personal communication, JF Ludvigsson, 4 Dec 2024).

The private expenditure that takes up the remaining 14% of the total health expenditure (92%) is largely attributable to household out-of-pocket payments [23]. Out-of-pocket payments are mostly from patient fees, co-payments, or unreimbursed services or medications (most medications are subsidized). Each region sets copayment rates for outpatient visits and hospital stays, leading to some variation across the country. The maximum amount to pay for outpatient care per 12 months (about 1,450 SEK, rate of 2025) is set at the national level. Child and maternity care, dental care for individuals < 20 years, and some care for individuals > 85 years are exempt from patient fees. Pharmaceutical and dental benefits are also determined at the national level. High-cost protections apply to both prescribed medications and dental care. Medications covered by the National Drug Benefit Scheme become free of charge when their total costs per 12 months exceed 2,900 SEK (~ 259 Euro, rate of 2025). In contrast, compensation will be provided for dental expenses exceeding 3,000 SEK (~ 261 Euro) annually for individuals aged ≥ 24 years. A small number of prescribed medications, including contraceptives for females < 21 years (or < 25 years in some regions, e.g., Skåne and Blekinge) and insulin are free of charge in Sweden.

Voluntary health insurance (VHI) is highly marginalized in Swedish healthcare (only 0.6% of the total health expenditure in 2022) [23]. A notable increase in the insured population, particularly within high socioeconomic groups, has been observed over the past decade. Most (over 90%) Swedish VHI plans are group-based and financed by private employers, primarily serving as supplementary coverage for expedited access to private healthcare providers [25]. However, because of the negative implications for horizontal equity, VHI is highly regulated in terms of its coverage of care providers and services.

## Delivery of services

### Primary care

Primary care is non-hospital-based outpatient care for all patient groups, regardless of age or underlying conditions. The scope of primary care includes managing planned and unplanned visits within general medicine, along with a focus on rehabilitation, health promotion, and disease prevention. Team-based primary care is practiced in Sweden. Primary care centers typically have 40–50 employees, with about five general practitioners. The other employees are a mix of other professional backgrounds (e.g., nurses with different specializations, physiotherapists, assistant nurses, and administrators). Physicians in primary care are specialists in family medicine and, when needed, channel patients to other specialized care. There is a shortage of general practitioners [18], with few primary care centers meeting the national benchmark of a maximum 1100:1 patient-to-regular physician (*fast läkarkontakt*) ratio.

In 2023, among every 1,000 residents, there were 3,478 physical visits to primary care [26]. For planned visits, primary care physicians diagnose, treat, and monitor highly prevalent chronic diseases and psychiatric disorders (e.g., hypertension, type 2 diabetes, asthma, depression, and anxiety) as well as diagnose and refer acute or severe diseases (e.g., angina pectoris, severe infections, and cancers). When collaborating with municipal care, they are also consulted on maternal/antenatal care and well-baby clinics. Primary care physicians are also involved in rehabilitation and long-term care provided by municipalities, even though they are de facto employed by the regions. At the same time, primary care centers respond to unplanned visits that do not require organ-specific expertise. They significantly contribute to regional developmental work and disaster preparedness, but their involvement in clinical research is less extensive.

Some 46% of the primary care centers in Sweden are run by private providers, subject to the same regulations and reimbursement mechanisms as publicly operated primary care centers. In 2023, about 39% of the total costs for primary care were generated by private providers and funded regionally [27]. Primary care centers have different names, from “care center (*vårdcentral*)” or “health center (*hälsocentral*)” to “family physician’s office (*familjeläkarmottagning*)”. There are also “on-call centers (*jourcentral*),” where primary care centers collaborate to offer non-specialized emergency care.

### Specialized care

Specialized care covers somatic and psychiatric conditions and is delivered in outpatient or inpatient settings. SALAR reports 1,664 outpatient visits (of which 5 are daycare visits) and 121 hospital admissions for somatic care per 1,000 residents in 2023. There were 482 outpatient visits and 10 hospital admissions per 1,000 residents for psychiatric care [26].

Following Sweden’s initiative to curb hospital admissions, day surgeries have increased almost three-fold since 2006, reaching 2.1 million visits in 2023. Conversely, hospital-based surgeries and average care lengths have steadily decreased (0.5 million episodes and 5 days in 2023) [26]. In turn, hospital beds have also reduced to 1.9 for every 1000 residents in Sweden (2022 data) (Fig. 3), which is among the lowest across the EU [28] and has resulted in bed occupancy rates surpassing 100% in some regions. Originating as a cost-containment strategy in response to Sweden’s 1990s economic downturn, this trend may indicate progress in clinical practice (including specialized elderly care and functionally impaired patient care, as well as expanded extra-hospital care provision); however, it presents certain drawbacks. The unplanned 30-day readmission rate among the elderly, as an indicator of surgical care quality, has been largely unchanged since 2010 [29]. The decrease in hospital beds could also result from constraints on human resources. In turn, the capacity shortage may aggravate the emergency departments’ crowding [29].

Although the Swedish healthcare system is decentralized, highly specialized care is only centrally offered at a maximum of five healthcare units (most often university hospitals). Regions are specially required by the Swedish National Board of Health and Welfare (*Socialstyrelsen*) to cooperate in such care [10]. Selected neonatal surgical procedures, including those for Hirschsprung’s disease, are provided exclusively at several university hospitals. Centrally offered care also applies to heart transplantations, gender dysphoria surgeries, cochlea implants, and treatments of osteogenesis imperfecta. The purpose of centralizing these interventions is to increase knowledge of the conditions through hands-on experiences and thus to improve quality and patient safety. Decisions on providers of such highly specialized care are made exclusively by the National Board of Health and Welfare.

Private providers also operate in specialized care and are subject to the same regulations and reimbursement mechanisms (if accredited by the local government) as public providers. Private providers in specialized care share comparably less of the market than primary care providers. In 2023, region-funded private care took up 8% of the total costs for specialized somatic care and 10% of the total costs for psychiatric care [27]. Private providers play their most important role in dental care, accounting for 72% of the total consumption [30].

### Emergency care

Emergency care includes pre-hospital paramedic care and hospital-based care for urgent or intensive medical conditions. Responsibility for emergency care is shared between the some 60 emergency hospitals and the 7 university hospitals (additional hospitals also care for critical or complicated conditions). Efficiency is greatly emphasized. Diagnosis, treatment, and triage start even before patients arrive at the hospital, and telehealth systems for specialist reading of ambulance electrocardiograms are in place nationwide. Emergency medical transport is facilitated by ground and air vehicles, with cross-border treatment (such as in Norway) available as needed. The paramedic team includes practitioners authorized to prepare and administer medications, and at least one registered nurse trained in pre-hospital care is mandated. The emergency department is staffed with, among other practitioners, nurses and attending specialist physicians (on-roster or in emergency medicine) [29]. The annual number of emergency care visits is approximately 1.9 million, among which some 30,000 have psychiatric conditions as the main complaint [29, 31]. However, emergency care resources are scarce in Sweden. Every 100,000 residents have to share a mere 5.7 intensive care beds on average (2023 data), among the lowest across the EU or OECD countries [32]. Moreover, despite the efforts to speed up emergency care and enhance access equity, emergency care in Sweden still faces increasingly prolonged waiting times and a longer traveling time in less-populated regions [29]. The growth in waiting time results from a compounded impact of an aging population and a general shortage in human and physical resources at different levels of care (e.g., the spill-over effects from unmet needs in primary care or deficiency in hospital beds).

From 2020 to 2022, access to and delivery of healthcare had changed due to COVID-19. For instance, during 2020, non-COVID-19 healthcare decreased by about 10% in many areas in Sweden [33].

## Health outcomes and quality of care

Sweden has a healthy population with healthcare outcomes comparable to the EU and OECD (Figs. 1 and 3) [5]. In 2022, the life expectancy was 83.5 years, with a very low standardized avoidable mortality rate (Fig. 1). The crude prevalence rates of type 2 diabetes, cardiovascular disease, and mental disorders were similar to those in comparable countries (Fig. 1). Sweden has a very low use of antibiotics in primary care, suggesting a relatively high awareness of care safety (i.e., antimicrobial resistance) among primary care professionals (Fig. 3). Thirty-day survival rates after incident acute myocardial infarction are exceptionally high within the OECD, while cancer mortality rates are exceptionally low (Fig. 3) [34].

### The perspective of the public/patient experience

The most recent survey by the Swedish Agency for Health and Care Services Analysis reported that about 90% of respondents in Sweden experience consistently kind and respectful treatment within the healthcare system [35]. However, only about 64% of the Swedish respondents reported participation in healthcare decisions at primary care centers (compared to 79% in similar countries), while approximately 60% indicated insufficient consultation time with their physicians (Fig. 3) [35]. Moreover, patients of different ages, sex, or socioeconomic status may not have been treated equally. For example, individuals of lower educational backgrounds experience less person-centeredness in the healthcare provided [35], and they are less satisfied with healthcare overall (54% satisfaction compared to 74% for the population average) (Fig. 3).

## Access to medical products

In Sweden, the state plays a central role in regulating pharmaceuticals, consumables, and medical devices (via the Medical Products Agency, *Läkemedelsverket*), guiding their clinical use (via the National Board of Health and Welfare), and determining products’ subsidies with indications (i.e., the benefit scheme, via the Dental and Pharmaceutical Benefits Agency, *Tandvårds- och Läkemedelsförmånsverket*,* TLV*). The regions provide local recommendations for using products in primary and outpatient care settings (by the formulary committees, *läkemedelskommittér*). TLV collaborates with regions (sometimes represented by the Council on New Therapies, *NT-rådet*, for pharmaceuticals, and the Medical Technology Product Council, *MTP-rådet*, for medical devices) to negotiate pricing and introduce new or expensive medical products.

During the fiscal year 2023, 41.4 billion SEK (~ 3.6 billion Euro including patient fees) was spent on products covered by the benefit scheme (about 65% of the total pharmaceutical expenditure), and 13.4 billion SEK (~ 1.2 billion Euro or 20% of the total expenditure) for those used in inpatient care [36]. The regions are the sole funding source for pharmaceuticals administered during inpatient care. For covered pharmaceuticals prescribed and dispensed in outpatient care or retail pharmacies, regions remain the official payers. However, a considerable proportion is covered by targeted state grants (e.g., 70% of the forecasted cost in 2023) [37]. Despite the comprehensive coverage, patients still bear about 12% of the total pharmaceutical expenditure. Expenses are categorized as over-the-counter medications (approximately 9%) or non-reimbursable medications, the latter encompassing instances where the prescribed indication lacks reimbursement or the patient opts for a brand-name drug despite generic availability [38].

## Healthcare workforce

As of 2022, about 46,840 physicians were registered in Sweden. Of these, 33,386 (71.3%) are specialists, including those practicing family medicine, and the remaining are either in training or have chosen to work without a specialty [39].

Excluding administrative staff, the largest staff groups in Swedish healthcare are registered nurses (*n* = 132,848) and assistant nurses (the exact number is unknown due to the absence of licensing for this latter group; however, the government agency *Statistics Sweden* suggests 129,600 [40]). Other large groups of staff include psychologists (*n* = 11,736), midwives (*n* = 8,930), and biomedical analysts (*n* = 10,324) [39]. Most staff work in specialized somatic care (56%), followed by equal shares in primary care (13%) and specialized psychiatric care (12%).

In 2023, the estimated number of full-time healthcare practitioners exceeded 200,000, though these statistics’ precision may be limited. A majority of practitioners in municipal healthcare are assistant nurses and care assistants (about 89%), while the estimated number of registered nurses is 17,000 (about 10%) [41].

Most staff have permanent employment, but about one in every eight are paid hourly. Temporary employment is particularly common among assistant nurses, representing approximately 16% of regional and 10% of municipal employees, according to the most recent (2018) data [41, 42].

Numerically, Sweden has a similar registered nurse-to-population ratio as in many other EU countries (registered nurses/1000 inhabitants: Sweden 10.9; Norway 15.7; Finland 14.1; Germany: 12.0, Denmark 10.4, Fig. 3) [43]. Sweden has a somewhat higher number of practicing physicians (4.4/1000, compared to the EU average of 4.0, 2021 data) (Fig. 3). However, in contrast with other countries, Swedish statistics also include physicians in training and those on parental and sick leave. In recent years, the proportion of foreign-trained physicians has increased in Sweden and is currently about 30% [5], higher than in almost all the EU countries except Ireland. Physicians in Sweden tend to be more equally distributed across regions than in most other OECD countries [5].

There is a shortage of healthcare staff in the public care sectors of almost all regions. This shortage is especially evident for medical specialists (such as general practitioners, specialist nurses, psychologists, and dentists) and technicians (such as biomedical analysts) [10]. The main attributes of the deficit are thought to be the underlying growth of the Swedish population, paired with an undersized vocational training of nurses and primary care physicians [44]. These factors have created a negative feedback loop characterized by stressful work environments, inconvenient working hours, competition with private sectors, and a growing demand for histopathological analyses. Staff shortage is particularly severe in rural areas where healthcare providers have to rely on temporary practitioners to fulfill their responsibilities [45]. Hopefully, the regions have now prioritized the supply of medical specialists. The number of residents in specialist training has increased by over 12% from 2019 to 2023, suggesting that the workforce shortage may be alleviated over time [18].

## Health information system

Sweden has a long history of digitalization in healthcare, encompassing systems for patient charts, quality registers, and administration. In Sweden, healthcare providers are mandated to report patient information and care details to national registers. However, information technology (IT) systems are often divided and operate in parallel, even for similar data. For technical and legal reasons, including patient confidentiality protection under the Patient Data Act (*Patientdatalagen*, SFS 2008:355), information communication across IT systems is limited [46]. This situation may improve under the state government (represented by the e-Health Agency, *E-hälsomyndigheten*) and the SALAR’s joint vision for developing digital health. Efforts have been made to allow patient records and prescription histories to be shared across different providers, specifically via the National Patient Overview system (*Nationell patientöversikt (NPÖ*, only for public care providers) and the National Medication List (*Nationell läkemedelslista*) [47].

In a 2020 survey of 2500 Swedish adults conducted by the American Commonwealth Foundation, Sweden’s healthcare system demonstrated above-average performance in digitalization [36]. Almost all documentation in Swedish healthcare is digital. Patients can read their patient charts through the website 1177.se.

### Opportunities for medical research

Sweden is particularly well suited for medical research for many reasons. One important reason is that universal healthcare facilitates population-representative observational research. With nearly equal opportunity to access healthcare, prospective routine registration of life and care events, and a unique personal identity number that enables linkages across different registers, generations of residents can be followed up throughout their lifespan. In addition, given that fewer people forgo healthcare due to financial constraints compared to other countries [35], the observed socioeconomic inequalities on health status would be, to a lesser extent, biased by the accessibility to care.

A large share of Swedish healthcare data is documented in nationwide registers [48–54] and dedicated quality registers [17]. Until now, the National Patient Register [52] has not covered primary healthcare visits, however, a governmental committee is presently reviewing the necessity of its integration.

## Recent transformations in healthcare

### Knowledge-driven management within Swedish healthcare

For many years, Swedish healthcare has been characterized by national guidelines, quality care registers [17], systems for evidence-based medicine (e.g., The Agency for Health Technology Assessment and Assessment of Social Services (Table 1)), and knowledge-sharing between specialists from different regions. That said, most guideline writing has traditionally been decentralized and done on the regional level. In 2010, the Swedish government established regional cancer centers where evidence-driven management was emphasized even more. This became a model for a formalized “knowledge-driven” management system in 2018, including non-cancer medicine. The word “knowledge” in the project name and the project’s relation to the well-established concept of evidence-based practice remain undefined. The project’s primary focus is standardizing care protocols through the consolidation of regional guideline development within joint committees. As of 2024, 26 national program groups focusing on, e.g., living habits, gastrointestinal diseases, rare diseases, and surgery had been established, including national collaboration groups hosted by SALAR.

In April 2024, the Minister for Health and SALAR signed an agreement, forming a guideline-writing partnership between the main state authorities, regions, and municipalities. Government agencies involved in this work include the Public Health Agency (*Folkhälsomyndigheten*) and the National Board of Health and Welfare. The support is delivered as part of an agreement with SALAR, the members’ organization of regions, and municipalities in Sweden [55].

### Shifting towards “good quality, local healthcare”

Since 2018, Sweden launched a vision of a shift from hospital-based care to more localized settings, termed “Good quality, local healthcare” (*“God och nära vård”*) as part of the new Healthcare Act (SFS 2017:30, sometimes called the Health and Medical Care Act or the Health and Medical Services Act) [56]. The core values are person-centered, coordinated, proactive, and co-created care. Despite the name, this approach is not bound to primary care. It does not introduce a new level of care but rather strengthens the collaboration between existing caregivers, i.e., across professional and organizational borders, to provide services that correspond to the growing number of individuals with complex needs. This approach mainly involves primary care centers, specialized outpatient clinics, and municipal home-based patient care. According to the Ministry of Social Affairs, *“Good quality*,* local healthcare”* is designed to improve care for the elderly and those with complex needs, including individuals receiving care and social services in different settings and those with significant mental health requirements [57]. 

The Healthcare Act (SFS 2017:30) mandates that care be provided in proximity to the patient, with a right to medical evaluation within three days [56]. Besides timeliness in medical evaluation, more emphasis is placed on continuity of care. This model implies strengthened collaboration across the regions and municipalities. The state government has allocated specific funding to the regions to develop high-quality, local healthcare. Part of this investment mandates the development of primary care in sparsely populated areas, especially rural ones.

The transition to *“Good quality*,* local healthcare”* is facilitated by digital healthcare. As part of the reform, the development of digital infrastructure has allowed some care providers, particularly private ones, to serve people beyond their registered regions. In 2022, there were 6 million digital visits in Sweden. Digital visits made up 1 in 7 primary care visits that year. There was a notable increase in the adoption of digital healthcare services during the COVID-19 pandemic [33]. It supported the delivery of essential care (e.g., video-based consultation and patient follow-up) and facilitated the communication of health information (e.g., communicating valid information about the pandemic to the public) [58].

From the health system’s perspective, *“Good quality*,* local healthcare”* is expected to control costs through more efficient use of healthcare resources [57]. It also aligns with the goal of person-centeredness by increasing the accessibility of care and patient involvement in healthcare decisions [57].

## Discussion

Healthcare responsibilities in Sweden are shared among the national government, the 21 regional authorities, and the 290 municipalities. The system provides universal access to needed care and is predominantly tax-funded, while the completely private, i.e., voluntary insurance-driven care, is rare.

From an international perspective, Swedish healthcare has several strengths; namely, its universally accessible, tax-funded structure minimizes cost-related healthcare avoidance. Co-payment and out-of-pocket payments are low. Overall, people’s health in Sweden is good. Sweden boasts a globally leading healthcare system, demonstrably effective in preventing avoidable mortality (Figs. 1 and 3), and showing high post-treatment survival rates for conditions such as cancer and stroke [22]. Life expectancy rates are high, while infant and under-five mortality rates are exceptionally low [6]. Hospital admissions for many chronic diseases have decreased in the past few decades.

Since 2011 (when elderly care and care of people with functional impairment were included as healthcare, pushing the healthcare share of GDP up by 2%), total health expenditure as a share of GDP has been hovering at about 10.8% (an additional peak in expenditures was noted during the COVID-19 pandemic) [21]. In 2022, this proportion exceeded the EU average by approximately 2% [10].

However, the country does poorer on continuity and coordination of healthcare. There is still some horizontal inequity in Swedish healthcare on, for instance, cancer detection and treatment [59–61]. Young people and people with foreign backgrounds have a more negative opinion of healthcare than other age groups [35]. Although there has been a reduction in waiting times for specialized care [36], primary care access has deteriorated, and persistent issues of care continuity remain within the Swedish healthcare system. Long waiting time has been a major source of patient dissatisfaction within the Swedish healthcare system for many years [35].

Fragmentation in the Swedish healthcare system is reflected in many interdependent aspects, such as lack of continuity in care and coordination in healthcare governance and IT systems. Geriatric patients and those with multiple comorbidities frequently have numerous caregivers and physicians. For instance, a patient with rheumatoid arthritis may regularly see a rheumatologist (or, in some regions, an internist), but with parallel visits to an ophthalmologist for concomitant uveitis and a primary care physician for complaints unrelated to the underlying rheumatoid arthritis. Analogously, a patient with inflammatory bowel disease might undergo routine monitoring by a gastroenterologist, consult a rheumatologist for spondyloarthritis, seek a primary healthcare physician for migraine, and additionally, consult other specialists (e.g., a gynecologist) as needed. Each of these care chains may function well but are not always coordinated. Reasons for the lack of coordination include low proportion of general practitioners, separate IT systems (such as those for electronic patient charts), privacy laws obstructing coordination, and compensation systems that do not award coordination.

The Swedish healthcare system confronts several challenges, notably crisis preparedness and the widening disparity in healthcare access [62]. One study highlights a link between precarious employment and increased cardiovascular risk [63]. However, Sweden is one of the very few countries where at least half the population aged ≥ 65 years meet the WHO recommendations on physical activity [5], has one of the lowest adult smoking rates in Europe [5], and a low alcohol consumption (7.5 Liters per adult per year) [5] (Fig. 1), signifying the efficacy of its preventative healthcare programs.

Sweden’s accession to NATO implied a need for the healthcare system to be prepared for crises. Although Sweden had a lower excess mortality during the peak of the COVID-19 pandemic (i.e., during 2020 and 2021) than other European countries [33], fatalities were predominantly among those aged ≥ 70 years [33]. The surge in acute needs for healthcare revealed weaknesses in the Swedish healthcare system, such as deficiencies in hospital beds (i.e., lower than in most other EU or OECD countries, Fig. 3) and nursing staff [28, 43]. Fragmentation in governance, in which regionally employed physicians were less involved in planning municipality-organized elderly care, significantly harmed vertical equity (e.g., not prioritizing individuals with higher frailty) during the pandemic. Each region has a civil servant on call (*”tjänsteman i beredskap”*) as well as a special healthcare leadership (“*särskild sjukvårdsledning”*) for such crises. In addition to crisis preparedness in resources and organizations, Sweden must prepare legislatively to allow more effective and efficient responses to potential crises [33].

Sweden’s approach to meet future challenges emphasizes evidence-driven healthcare management and “*Good quality, local healthcare*”, reflecting a tripartite commitment from national, regional, and municipal levels to enhance accessibility. Sweden also needs to continue the effort to improve continuity. With limited primary care resources and surging healthcare demands, a review of priorities is required. It is unclear whether primary care should focus on prevention, treatment, or simply availability for any issue. Nowadays, Swedish primary care focuses disproportionally on availability based on patient choice rather than treating patients according to their needs.

This study has some strengths and limitations. We used a variety of sources for the article, including extensive clinical expertise. We reviewed the structure and funding of Swedish healthcare and put that into perspective—both compared to other countries and against background factors that might promote or hinder good health in Sweden.

The momentary nature of certain information is among the limitations. Changes in laws and regulations which affect healthcare management and the structure of Swedish healthcare may hinder appropriate conclusions. However, our study should be regarded as a serious attempt to describe the core components of the Swedish healthcare system.
